# A Genome-Wide Immunodetection Screen in *S. cerevisiae* Uncovers Novel Genes Involved in Lysosomal Vacuole Function and Morphology

**DOI:** 10.1371/journal.pone.0023696

**Published:** 2011-08-30

**Authors:** Florante Ricarte, Rosa Menjivar, Surya Chhun, Tattika Soreta, Lisa Oliveira, Teli Hsueh, Maribeth Serranilla, Editte Gharakhanian

**Affiliations:** Department of Biological Sciences, California State University Long Beach, Long Beach, California, United States of America; Institute of Developmental Biology and Cancer Research, France

## Abstract

Vacuoles of yeast *Saccharomyces cerevisiae* are functionally analogous to mammalian lysosomes. Both are cellular organelles responsible for macromolecular degradation, ion/pH homeostasis, and stress survival. We hypothesized that undefined gene functions remain at post-endosomal stage of vacuolar events and performed a genome-wide screen directed at such functions at the late endosome and vacuole interface – *ENV* genes. The immunodetection screen was designed to identify mutants that internally accumulate precursor form of the vacuolar hydrolase carboxypeptidase Y (CPY). Here, we report the uncovering and initial characterizations of twelve *ENV* genes. The small size of the collection and the lack of genes previously identified with vacuolar events are suggestive of the intended exclusive functional interface of the screen. Most notably, the collection includes four novel genes *ENV7*, *ENV9*, *ENV10*, and *ENV11*, and three genes previously linked to mitochondrial processes – *MAM3*, *PCP1*, *PPE1*. In all *env* mutants, vesicular trafficking stages were undisturbed in live cells as assessed by invertase and active α-factor secretion, as well as by localization of the endocytic fluorescent marker FM4-64 to the vacuole. Several mutants exhibit defects in stress survival functions associated with vacuoles. Confocal fluorescence microscopy revealed the collection to be significantly enriched in vacuolar morphologies suggestive of fusion and fission defects. These include the unique phenotype of lumenal vesicles within vacuoles in the novel *env9Δ* mutant and severely fragmented vacuoles upon deletion of *GET4*, a gene recently implicated in tail anchored membrane protein insertion. Thus, our results establish new gene functions in vacuolar function and morphology, and suggest a link between vacuolar and mitochondrial events.

## Introduction

A hallmark of eukaryotic cells is their ability to meticulously organize and compartmentalize tasks within membrane bounded organelles. An example of such a specialized organelle is the dynamic lysosome. The lysosome is involved in vital cellular functions including macromolecular degradation, receptor down regulation, pH/ion homeostasis, and stress survival [1]–[4]. The yeast vacuole is functionally analogous to the mammalian lysosome and serves as an elegant genetic model for studying lysosomal function, biogenesis, and trafficking. Yeast genes involved in these processes often have conserved orthologues in higher eukaryotes [5], [6].

Three conserved pathways are responsible for delivery of cargo to the vacuole. The biosynthetic pathway involves sorting of newly synthesized vacuolar proteins away from the secretory pathway in trans Golgi and their vesicular delivery to the vacuole [7]–[11]. The endocytic pathway involves the vesicular delivery of external and cell surface components through the early endosome to late endosome, and through the maturation product of late endosome – the multivesicular body (MVB)—to the vacuole [1], [2], [12]. The conserved macroautophagic and the yeast-specific cytoplasm-to-vacuole targeting (Cvt) pathways involve the transport of cytoplasmic components directly to the vacuole through vesicular delivery [4], [13]–[16]. All three trafficking pathways involve a final step of heterotypic membrane fusion at the vacuole. Furthermore, the vacuole itself is a dynamic organelle which undergoes homotypic fusion/fission events during budding and in response to environmental stress and nutrient levels. Due to limited available mutants, heterotypic fusion remains less understood than homotypic fusion. Nonetheless, all membrane fusion/fission events at the vacuole remain of intense interest [17], [18]. Several models have been presented to elucidate late endosome to lysosome trafficking, including endosomal maturation, direct fusion of endosome to lysosome, and carrier transport [19], [20]. Two models that support direct fusion include the “kiss and run” model where fusion and fission of endosomes and lysosomes occur to exchange cargo, and the “hybrid” model where a hybrid organelle is formed by heterotypic fusion of late endosome and lysosome [21]–[24]. The continued persistence of multiple models is largely due to the limited gene functions identified at the final stages of lysosomal delivery and processing. We are interested in exploring such gene functions in yeast.

As in mammalian cells, the biosynthetic pathway delivers the bulk of the resident hydrolytic enzymes of the vacuole including the well characterized carboxypeptidase Y (CPY). CPY is synthesized as an inactive zymogen and undergoes core glycosylation in the rough endoplasmic reticulum to form p1CPY precursor. It is then transported through Golgi compartments where it is further glycosylated into p2CPY form. In trans Golgi, p2CPY is sorted from the secretory pathway by its receptor, Vps10p, and is delivered to the late endosome [11], [25]. At the late endosome, p2CPY is uncoupled from its receptor and is delivered to the vacuole where it is processed into mature CPY in a Proteinase A (PrA) dependent manner. Vps10p receptor is recycled back to trans Golgi via the retromer complex and is engaged in repeated cycles of p2CPY sorting and delivery at the trans Golgi and late endosome interface. Several laboratories have reported genetic screens for yeast mutants defective in the CPY pathway including *pep*, *vps*, and *vam* mutants [26]–[30]. These mutant collections generally secrete p2CPY. We hypothesized that in mutants exclusively defective at post-endosomal stage of vacuole delivery/function, Vps10p would retain its ability to cycle back to trans Golgi and direct p2CPY to late endosomes. Based on this hypothesis, we developed a novel immunodetection screen for mutants that internally accumulate p2CPY at the late endosome and vacuole interface – *env* mutants [31]. In the current study, we modified and applied the approach on a genome-wide scale using the MAT-α haploid deletion strain library, which uncovered 12 *ENV* genes. This study ascribes new vacuolar processing and morphology functions to several formerly characterized genes and uncovers four novel *ENV* genes, *ENV7, ENV9, ENV10, and ENV11*.

## Results

### Genome-wide immunodetection screen

**Figure 1:**
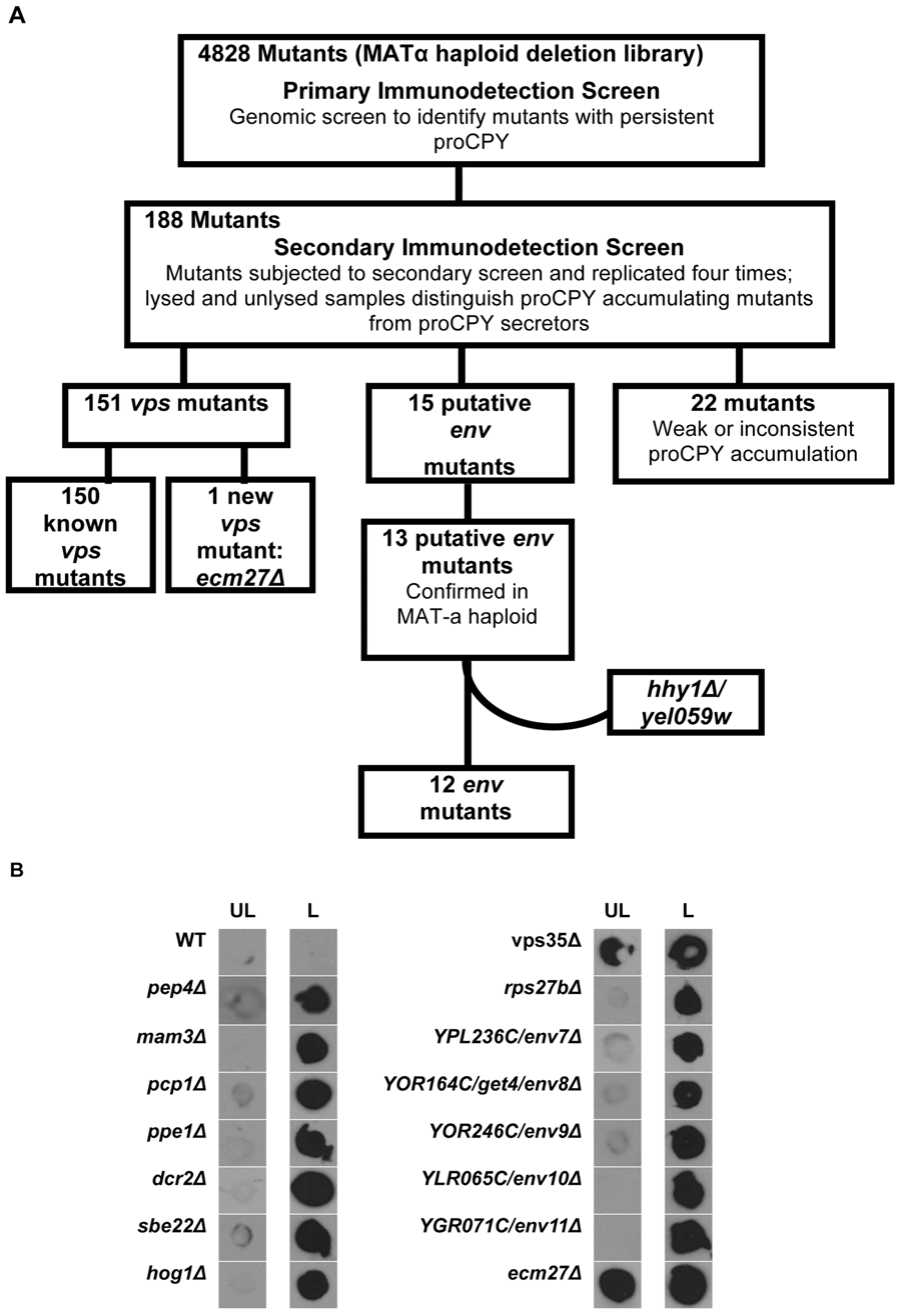
Genomic immunodetection screen- flowchart and results. **A.** Flowchart of screen strategy and results as detailed in Materials and Methods section. **B.** Repeated immunodetections with mAb specific to pro region of CPY confirmed twelve mutants that internally accumulate pro-CPY (*env* mutants) and one new pro-CPY secreting mutant (*vps* mutant), *ecm27Δ*. The BY4742 (WT), *pep4Δ* and *vps35Δ* strains are included as controls (L = lysed, UL = unlysed).

Previously, we reported a novel colony Immunodetection genetic screen for internal accumulation of precursor CPY [31]. Here, we modified and applied the screen at a genome-wide level to the MAT-α haploid deletion strain library in two stages (Figure 1). In the primary immunodetection screen, we uncovered 188 mutants that indicated any proCPY presence above wild type levels in lysed patched samples. Uncovered strains were further pursued in a secondary screen of lysed and unlysed patched strains to confirm persistent proCPY and identify mutants that specifically accumulate proCPY internally. The secondary screen was repeated at least four times for each of the 188 mutants and only the 166 mutants which gave persistent and strong proCPY signal in repeated experiments were pursued beyond the secondary screen. During primary and secondary screens, the strains were identified only by grid/plate number to minimize bias. The mutants were matched to systematic/gene names at the completion of the two stage screen. 151 of the 166 strains secreted proCPY and 150 of those were, in fact, previously identified *vps* mutants in the original mutant screens [27], [32] and the genome-wide screen of deletion mutants [30]. Additionally, one previously unreported *vps* mutant, *ecm27Δ*, was uncovered. Fifteen putative *env* mutants were identified at the end of the secondary screen stage. Since errors and extraneous mutations have been reported with the haploid deletion strain collection, MATa counterparts of the putative mutants were also assessed for *env* phenotype via patch immunoblots. Aside from the deletion mutant for proteinase A (PrA), the vacuolar hydrolase responsible for CPY maturation [33], thirteen *env* mutants were confirmed in their MAT-a counterparts, and their deleted genes were confirmed by PCR. One of the thirteen mutants, *hhy1/yel059w*, was also uncovered by us in a genome-wide screen for hygromycin B hypersensitivity. The ORF is designated as a “dubious ORF, unlikely to encode a protein” in the *Saccharomyces* Genome Database (SGD), and the reintroduction of the ORF does not complement the env or the hygromycin B hypersensitivity phenotypes and the deletion of the ORF does not result in either phenotype ([34], unpublished results). Thus, the responsible locus for the phenotype remains unmapped. Table 1 lists the 12 uncovered *ENV* genes and their annotated biological process, molecular function, and cellular component as gleaned from SGD (http://www.yeastgenome.org). The collection does not include any genes previously identified with vacuolar events. *DCR2* overlaps 34 bases of the *VPS38* C-terminus coding sequences. As such, it may signify a weak *VPS38* allele. A previous genome-wide study aimed at identifying endosomal transport factors conducted quantitative immunoblot assays to assess secretion of CPY in the deletion strain library [35]. Of the 12 *ENV* genes, *DCR2* was the only one consistently scored in the top 200 hits. However, since *DCR2* encodes a protein involved in ER unfolded protein response, it was pursued as part of the *ENV* collection. The uncovered twelve genes are not allelic to the loci implicated in *env1-env3* allelic mutants, and complementation assays in diploids of conditional *env4* and *env5* haploids and the non-isogenic deletion env mutants have been inconclusive ([31], unpublished results).

**Table 1:** Uncovered genes and their known biological processes, molecular functions, and cellular components (Saccharomyces Genome Database).

*ENV* Genes	Biological Process	Molecular Function	Cellular Localization
*MAM3 (YOL060C)*	Required for normal mitochondrial organization and cell Mn^2+^ homeostasis	Unknown	Vacuole membrane
*PCP1 (YGR101W)*	Required for normal mitochondrial organization, protein import into mitochondrion IM space, signal peptide process	Peptidase activity	mitochondrion
*PPE1 (YHR075C)*	Protein modification process; small subunit mitochondrial ribosome protein	Protein with carboxyl methylesterase activity	Soluble fraction
*DCR2 (YLR361C)*	ER unfolded protein response, protein aa dephosphorylation activity, pt of start for mitotic cycle	Hydrolase, phosphoesterase involved in down regulation of the unfolded protein response	Unknown
*SBE22 (YHR103W)*	Involved in the transport of Cell wall components (bud growth), functionally redundant with sbe2p	Unknown	Cytoplasm
*HOG1 (YLR113W)*	Hyperosmotic response, osmosensory signaling pathway regulation of RNA pol II transcription	MAP kinase activity	Cytoplasm, nucleus
*RPS27B (YHR021C)*	Cleavage of tricistronic rRNA transcript and maturation, translation, small ribosomal subunit assembly	Structural constituent of ribosome	Cytosolic small ribosomal subunit
*ENV7 (YPL236C)*	Unknown	Protein serine/threonine kinase activity	Fungal-type vacuole membrane
*GET4/ENV8 (YOR164C)*	Posttranslational protein targeting to membrane; protein insertion into ER membrane	Unknown	Cytoplasm
*ENV9 (YOR246C)*	Unknown	Oxidoreductase activity	Lipid particle
*ENV10 (YLR065C)*	Unknown	Unknown	unknown
*ENV11 (YGR071C)*	Unknown	Unknown	Nucleus

*VPS* Gene	Biological Process	Molecular Function	Cellular Localization
*ECM27*	Cell wall organization	Unknown	Unknown

GO TermFinder analysis displayed no significant enrichments. However, based on published information on *ENV* gene products, we note a few key groupings. Three *ENV* genes function in mitochondrial morphology and/or organization. *MAM3* was originally identified to be involved in mitochondrial morphology in a systematic study [36]. More recent studies reveal that it is an integral vacuolar membrane protein whose degrees of expression mark the degree of manganese toxicity [37]. *PCP1* encodes a rhomboid-like mitochondrial intramembrane serine protease that is essential for normal mitochondrial morphology and DNA maintenance [38], [39]. At least two functions have been noted for *PPE1*. Its gene product has been previously identified as a mitochondrial ribosomal protein [40] and also as a carboxyl methylesterase that inactivates the catalytic subunit of phosphoprotein phosphatase 2A (PP2A) [41].

Three *ENV* gene functions are associated with the endomembrane system. *SBE22* encodes a Golgi protein that functions in transporting components necessary for cell wall formation from the Golgi to the cell surface and aids in cell wall integrity [42]. *DCR2* encodes a phosphoesterase that acts as both a dosage-dependent cell cycle regulator [43] and a down regulator in the ER unfolded protein response [44]. During our studies, one uncharacterized *ENV* gene, *ENV8*, was identified by others as a member of the GET complex genes involved in tail-anchored protein insertion and named *GET4* [45], [46].

Two *ENV* genes are cytoplasmic residents. *RPS27B* encodes the protein component of the small (40s) ribosomal subunit and may be involved in the structural dynamics of the ribosome [47], [48]. *HOG1* has been extensively studied as the central node in the HOG signaling pathway involved in osmotic stress response. Hog1p is a mitogen-activated protein (MAP) kinase; under hyperosmotic stress it localizes to the nucleus and mediates upregulation of genes by phosphorylating transcription factors [49], [50]. Its mammalian homolog, MAP kinase p38, also functions in hyperosmotic and stress responses [51], [52].

**Figure 2:**
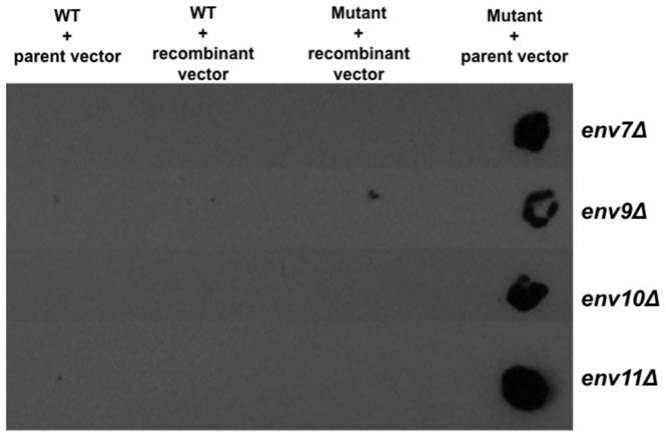
*ENV7*, *ENV9*, *ENV10*, *ENV11* ORF's complement *env* phenotype of their corresponding deletion mutants. The four strains deleted in each of the orphan genes were transformed with parent CEN vector or CEN vector containing the corresponding deleted ORF and its flanking 300–500 upstream and downstream sequences. Transformed cells were patched onto SM-URA plates for selection and were subjected to colony immunodetection with anti-proCPY specific mAb. WT(BY4742) transformed with parent or recombinant vectors containing each of the four ORF's were grown and processed in parallel as controls.

Lastly, four *ENV* genes are novel and have no previously established functions – *ENV7*, *ENV9*, *ENV10*, and *ENV11*. To confirm that the *env* phenotype in the four mutants is a result of the deleted genes, we constructed recombinant CEN vectors encoding each of the novel ORF's, introduced each into the corresponding deletion mutant, and assayed for phenotypic complementation by means of colony immunodetection (Figure 2). In each case, the mutant phenotype is rescued with the reintroduction of the corresponding ORF and its endogenous upstream/downstream regulatory sequences. Thus, *ENV7*, *ENV9*, *ENV10*, and *ENV11* are four novel genes whose individual deletion leads to internal accumulation of precursor CPY.

### Secretion and α-factor processing in *env* mutants

**Figure 3:**
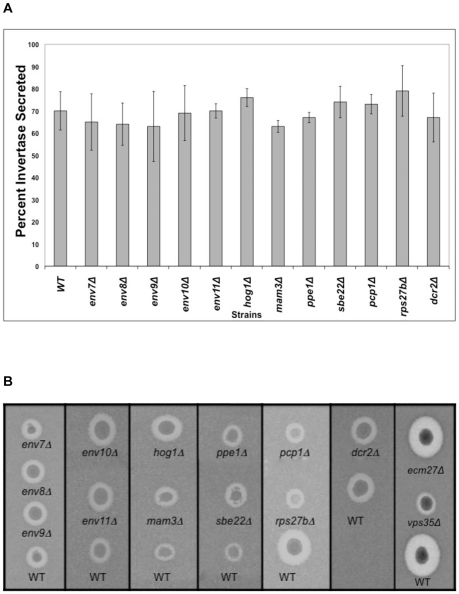
Invertase and active α-factor secretion in *env* mutants. **A.** A quantitative colorimetric assay for glucose presence was used to assess invertase secretion as detailed in Results. Secreted invertase is expressed as the percentage of extracellular invertase over total intracellular and extracellular .invertase. Bars indicate standard deviation (Two-Sample *t*-Test, N = 3, confidence interval 95%, *p*<0.05). **B.** Qualitative halo assays were used to assess active α-factor secretion by equivalent inocula of MAT-α *env* strains and isogenic WT onto a lawn of supersensitive MAT-a GPY-1796 cells. Each vertical column is a single plate with its WT control; visual alignment was achieved using Photoshop without alteration of experimental data.

Internal accumulation of p2CPY in putative *env* mutants could be conceivably due to blocks in the pre-endosomal stages of trafficking including secretion. To assess early trafficking events as well as trans Golgi to plasma membrane trafficking, we conducted quantitative invertase secretion and qualitative active pheromone secretion assays (Figure 3, A and B). When yeast is grown on sucrose as the sole carbon source, secreted invertase catalyzes the disaccharide to glucose and fructose for uptake through their corresponding permeases and can be quantitatively measured via a colorimetric assay for glucose. Such quantitative assays showed no statistically significant secretion defect in *env* mutants relative to the isogenic wild type (Figure 3A). Thus, secretion and all vesicular trafficking events shared between the vacuolar biosynthetic and secretion pathways remain intact in *env* mutants. As such, *ENV* gene deletions do not result in defects at ER/Golgi, intraGolgi, or Golgi/plasma membrane interfaces. The pheromone halo assay was used next to evaluate protein trafficking at the late endosome and trans Golgi interface in the *env* mutants as well as the new *vps* mutant, *ecm27Δ* by assessing protease dependent activation of α-factor in trans Golgi. Mature α-factor is a small peptide pheromone that arrests the cell cycle and cell growth of MAT-a yeast cells in preparation for mating [53]. It transits the early stages of vesicular trafficking as a pro-pheromone and is processed in trans Golgi in a protease dependent manner to its mature, active form prior to secretion. Several proteases that recycle between trans Golgi and late endosome have been implicated in pro- α-factor maturation and activation in trans Golgi including Kex1p, Kex2p, and DPAP-A [54]–[57]. Active pheromone is capable of arresting the cell cycle of a lawn of supersensitive MAT-a cells in halo assays. As intact secretion was confirmed by invertase assays, any defect in halo formation would indicate defects at trans Golgi and late endosome interface. This assay has been used repeatedly in our laboratory to assess both major and minor defects in α-factor secretion [34], [58]. From equivalent inocula, equivalent zones of growth inhibition relative to patch size were seen in *env* mutants, ecm27*Δ*, and the isogenic wild type in repeated experiments (Figure 3B). As expected, a severely compromised halo is seen in v*ps35Δ*, which lacks the Vps35p component of the retromer complex involved in retrograde trafficking from late endosome to trans Golgi. Due to their slower growth, *pcp1Δ* and *rps27bΔ* exhibited both smaller patches and halos relative to wild type. Thus, defects were not noted in vesicular trafficking stages leading to secretion nor in anterograde/retrograde trafficking at trans Golgi and late endosome interface in *env* mutants..

### Bulk endocytosis and vacuolar morphology in the uncovered mutants

**Figure 4:**
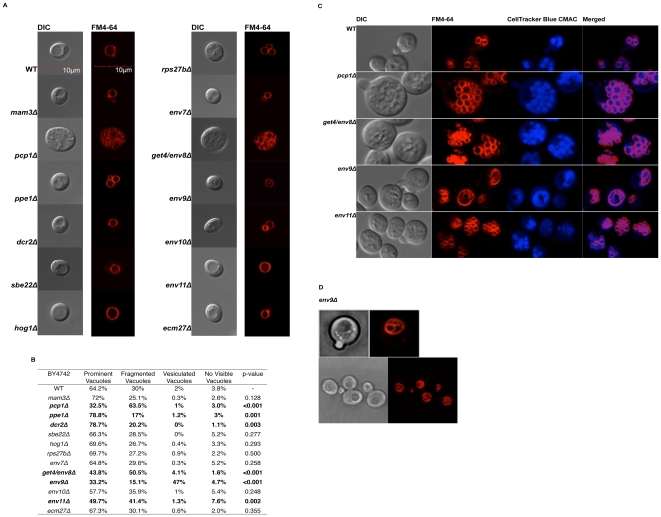
Vacuolar morphology of uncovered mutants. **A.** Logarithmically growing cells were stained with vital dye FM4-64 for 60 min and viewed using DIC optics and confocal microscopy. The predominant vacuolar morphology observed is shown. **B.** Percentages of observed vacuolar morphology phenotypes. For each strain, 300–400 non-budding cells were scored in randomized fields; raw data were subjected to Chi-squared analysis. Mutants showing statistically significant difference from wild type are in bold. **C.** Strains exhibiting abnormal vacuolar morphology were subject to costaining with vital dyes FM4-64 and CellTracker Blue CMAC. **D.** Representative DIC and fluorescent confocal images of FM4-64 stained *env9Δ.*

To assess the integrity of bulk endocytosis and vacuolar morphology, uncovered strains were stained with FM4-64 and viewed with DIC and confocal microscopy (Figure 4A); 300–400 cells were scored per strain for statistical analysis (Figure 4B). FM4-64 is a vital fluorescent styryl dye that embeds itself into the plasma membrane and eventually localizes to the vacuole membrane through the endocytic pathway. As such, it serves as both an endocytic marker and a vacuole membrane marker in live cells. In wild type cells, the vital dye localizes to the vacuole within 60 minutes of incubation at 30°C [59]. Since FM4-64 traverses the cell along the endocytic pathway, localization of the dye to the vacuole membrane requires intact vesicular trafficking at the interfaces of plasma membrane, early endosome, late endosome, and vacuole. We observed that FM4-64 localizes to vacuole membranes in all uncovered mutants, indicating intact bulk endocytosis. Vacuolar localization of FM4-64 in mutants with drastic vacuolar morphology defects was confirmed by costaining with the vacuolar dye Cell Tracker Blue (Figure 4C).

In the isogenic wild type BY4742 strain, the predominant vacuole morphology is 1–4 prominent vacuoles seen in 64% of cells, while 30% have fragmented vacuoles (Figure 4, A and B). The new *vps* mutant, *ecm27Δ*, displays a wild type vacuole morphology pattern and can be classified as Class A (Figure 4, A and B). Six of the 12 *env* deletion strains have statistically significant vacuolar morphology differences from wild type (Figure 4, A and B). *pcp1Δ and get4/env8Δ* strains exhibit predominantly fragmented vacuoles at 63.5% and 50.5%, respectively; *env11Δ* exhibits a significant increase in cells with fragmented vacuoles at 41.4%. Additionally, both *pcp1Δ* and *get4/env8Δ* cell sizes are significantly larger than that of wild type in the MAT-α background. The average size of the wild type cells observed is approximately 4 µm in diameter, whereas *pcp1Δ* and *get4/env8Δ* strains are approximately 8–10 µm in diameter. However, the larger cell sizes were not observed in the MAT-a counterparts (data not shown) and may be specific to MAT-α mating type. *ppe1Δ* and *dcr2Δ* exhibit statistically significant reduction in number of cells with fragmented vacuoles at 17%, and 20.2%, respectively. Lastly, the prominent vacuole morphology in the novel *env9Δ* mutant is one or more lumenal vesicles within the vacuole in 47% of scored cells in both mating types (Figure 4, A–C). The vacuolar morphology phenotype was complemented upon reintroduction of *ENV9* gene (data not presented). To our knowledge, lumenal vesicles have not been previously reported in vacuolar morphology studies. Thus, six of the twelve *env* mutants have vacuolar morphologies suggestive of vacuole fusion and fission defects.

### Growth of *env* mutants under various stress conditions

**Figure 5:**
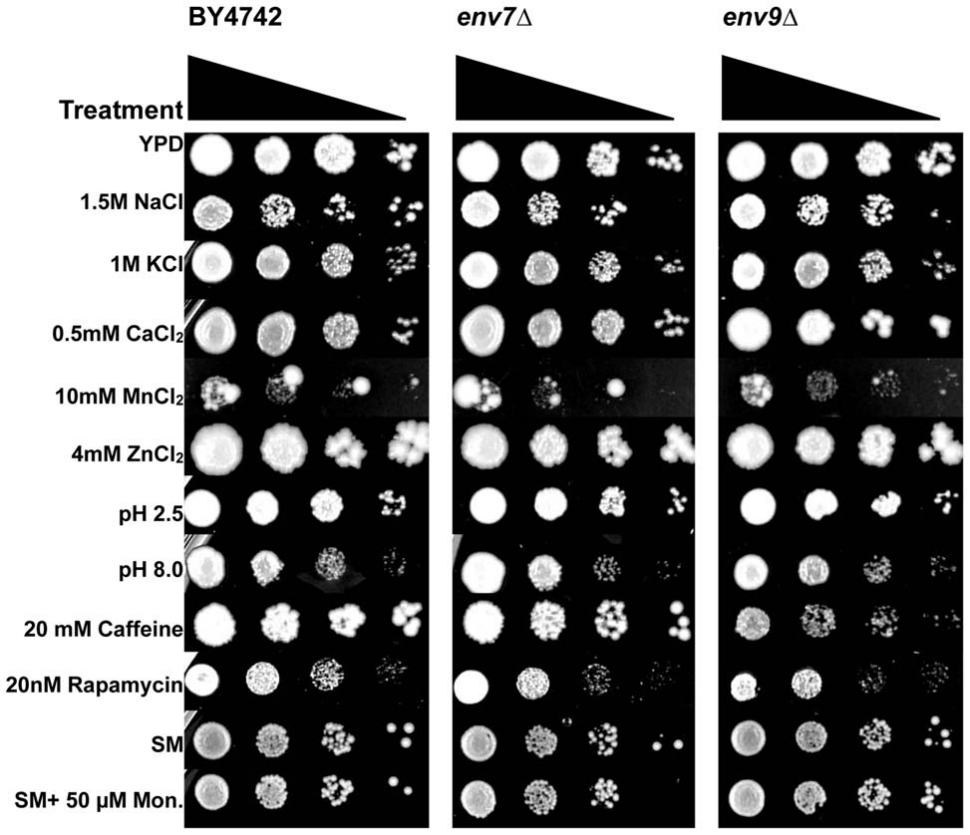
Growth characterizations of *env* mutants under various ion and drug conditions. Equivalent OD_600_ units of WT (BY4742) and mutants were stamped at multiple dilutions as specified in Materials and Methods. Wild type, *env7Δ* and *env9Δ* strains are represented in the figure; data for all mutants are presented in Table 2.

*env* mutants were assayed for pleiotropic growth defects under several drug, pH/ion and temperature conditions (Figure 5; Table 2). Growth characterizations were formulated based on previous studies of conditions that affect mutants defective in vacuolar trafficking and/or function. Since wild type growth was retarded under several tested conditions, mutant growth was scored relative to the isogenic wild type for each specific condition. Three strains show growth sensitivities to high salt suggestive of defects in osmoregulation, a vacuolar function. *pcp1Δ* and *rps27bΔ* are sensitive at 1.5 M NaCl, and *hog1Δ* is unable to grow at both 1.0 M and 1.5 M NaCl consistent with the established role of its MAP kinase product in osmoregulation [50]. *hog1Δ* is also sensitive to potassium and low pH.

**Table 2:** Growth characterizations of uncovered mutants under selective conditions.

BY4742	YPD	1.0 M Na^+^	1.5 M Na^+^	1.0 M K^+^	10 mM Mn^+2^	4 mM Zn^+2^	0.5 mM Ca^+2^	pH 8.0	pH 2.5	20 nm rapamycin	20 mM caffeine	SM+ 0.5% ethanol	SM + 50 µM monensin	YPD 15°C
WT	++	++	++	++	++	++	++	++	++	++	++	++	++	++
*env7Δ*	++	++	++	++	++	++	++	++	++	++	++	++	++	++
*get4/env8Δ*	++	++	++	++	++	++	++	++	++	++	++	++	++	−
*env9Δ*	++	++	++	++	++	++	++	++	++	++	+	++	++	++
*env10Δ*	++	++	++	++	++	++	++	++	++	++	++	++	++	−
*env11Δ*	++	++	++	++	++	++	++	++	++	++	++	++	++	−
*mam3Δ*	++	++	++	++	+++	++	++	++	++	+	−/+	++	++	++
*pcp1Δ*	++	++	−/+	++	−/+	++	++	++	++	−/+	−/+	++	++	−
*ppe1Δ*	++	++	++	++	++	++	++	++	++	++	++	++	++	++
*dcr2Δ*	++	++	++	++	++	++	++	++	++	++	++	++	++	++
*sbe22Δ*	++	++	++	++	−	++	++	++	++	++	−/+	++	++	++
*hog1Δ*	++	−	−	−	++	++	++	++	−/+	++	++	++	++	++
*rps27bΔ*	++	+	+	++	−	++	++	++	++	−/+	−	++	++	++
*ecm27Δ*	++	++	++	++	++	++	++	++	++	++	++	++	++	++
+++	stronger growth than WT													
++	WT growth													
+	weaker growth than WT													
−/+	significantly weaker growth than WT													
−	no growth													

*pcp1Δ*, *sbe22Δ*, and *rps27bΔ* exhibit severe sensitivities to manganese, while *mam3Δ* grows more robustly in presence of manganese relative to wild type. *mam3Δ* phenotype is consistent with previous studies that attribute increased manganese tolerance to *MAM3* deletion [37]. *mam3Δ*, *pcp1Δ*, and *rps27bΔ* also exhibit growth defects in presence of both rapamycin and caffeine. Growth sensitivities to both drugs are hallmarks of defects in Tor kinase complex 1 (TORC1) mediated signal transduction, and Tor1p kinase localizes to vacuolar membranes in living yeast cells [60].

*env9Δ* and *sbe22Δ* show growth sensitivities to caffeine but not rapamycin, suggestive of defects in cell wall integrity pathway. *sbe22Δ* has been previously identified as playing a significant role in cell wall formation [42]. Growth at 37°C and 15°C were also assayed. *env* mutants exhibit no growth sensitivities at 37°C (data not presented). Cold sensitivity is suggestive of compromised dynamics in large, multi-subunit complexes that render them “frozen” at low temperatures. The novel mutants *env10Δ* and *env11Δ*, and the only two mutants with predominantly fragmented vacuoles, *pcp1Δ* and *get4/env8Δ*, are cold sensitive (Table 2).

Lastly, monensin sensitivity has been linked to acidification defects in Golgi and post-Golgi compartments [61]. Recently, a genomic screen for monensin sensitivity (*mon* mutants) was heavily enriched for post-Golgi trafficking, which also included a subset of genes involved in vacuole biogenesis [61]. None of the *env* mutants were included in the *mon* collection, and our assays for monensin sensitivity did not uncover any growth defects (Table 2).

Thus, several members of *env* collection exhibit defects in vacuolar related stress survival that includes survival under rapamycin, caffeine, manganese, and osmotic stress.

### Steady state accumulation of p2CPY in *env* mutants

**Figure 6:**
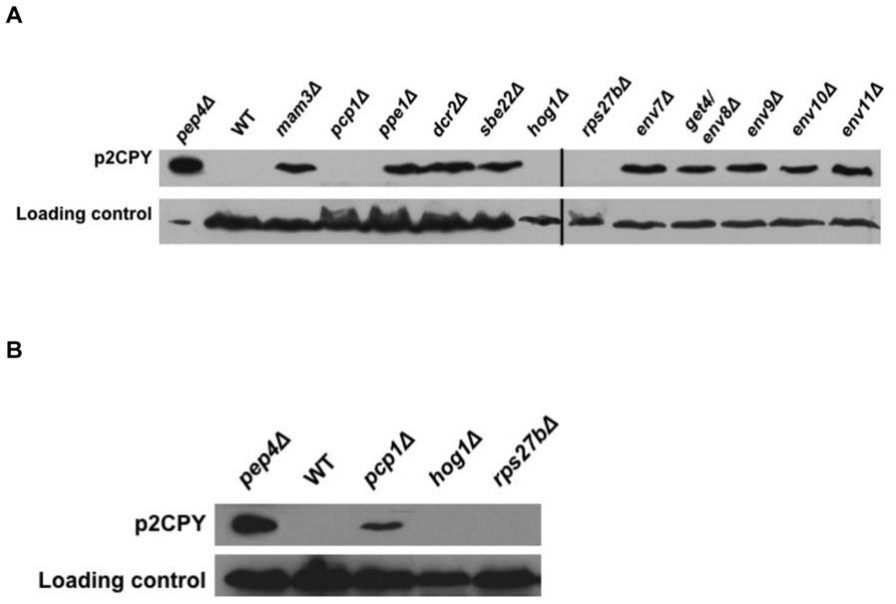
Steady-state accumulation of p2CPY in *env* mutants. Strains were grown in YPD overnight and were then diluted for further growth to logarithmic phase in YPD (A), or YPD+0.75 M NaCl (B). Cell extracts were normalized for protein content and subjected to western analysis. *pep4Δ* mutants are completely defective in CPY processing and a 1∶5 dilution of cell lysate was used as control for more equivalent visualization of signal. Membranes were probed with anti-proCPY mAb; hexokinase I was used as loading control and detected with pAb's. Two separate gels in A are marked by a vertical bar.

We performed western blot analysis of cell lysates to confirm the internal accumulation of p2CPY in *env* mutants using the pro-specific monoclonal antibody (Figure 6A). *pep4Δ* mutants are completely defective in CPY processing and were used as a positive control for p2CPY accumulation. Steady state persistence of p2CPY could be confirmed in nine out of twelve *env* mutants in repeated experiments. No proCPY species could be detected in *rps27bΔ*, *pcp1Δ*, and *hog1Δ* lysates under the initial growth and western blot analysis conditions used. The three mutants are unique in the collection for exhibiting growth sensitivities to high salt conditions (Table 2). Hence, the three strains were subjected to western analysis subsequent to logarithmic growth under 0.75 M NaCl, which was the highest NaCl concentration that supported WT and mutant growths in liquid YPD cultures. Following high salt treatments, persistent p2CPY could be reproducibly seen in *pcp1Δ*, but not in *rps27bΔ* or *hog1Δ* strains. The two strains were also analyzed for p2CPY accumulation in westerns following treatments to which each had shown growth sensitivities in our studies (Table 2), including potassium, manganese, and caffeine, but no persistent p2CPY was detectible (data not presented). Thus, within the resolution of our western analyses, steady state intracellular accumulation of the 69 kDa p2CPY could be confirmed for *mam3Δ*, *pcp1Δ*, *ppe1Δ*, *dcr2Δ*, *sbe22Δ*, *env7Δ*, *get4/env8Δ*, *env9Δ*, *env10Δ*, and *env11Δ*.

## Discussion

Here, we have described a yeast genome-wide screen designed to uncover mutants with exclusive defects at the late endosome and vacuole interface – *env* mutants. We report a collection of twelve *ENV* genes whose deletion results in defects in vacuolar function and morphology. A combination of quantitative invertase and qualitative active α-factor secretion assays reveal no secretion or vesicular trafficking defects prior to the late endosome. The bulk endocytic pathway is also intact in all *env* mutants as assessed microscopically. As the biosynthetic CPY-pathway and the endocytic pathway converge at the late endosome, intact endocytosis also implicates normal trafficking from late endosome to vacuole. Together, these data spatially map the defect observed in *env* mutants to the vacuole. Such defects would be expected to include homotypic and/or heterotypic fusion/fission defects at the vacuole, or vacuolar function defects such as macromolecular processing or stress survival. Indeed, *env* mutants exhibit such defects. First, based on the screen itself, *env* mutants show CPY processing defects. Second, half the collection exhibits vacuolar morphologies suggestive of fusion/fission defects including a novel morphology of vesiculated vacuoles in *env9Δ* – a novel mutant itself. Third, a partially overlapping group of five mutants exhibit severe defects in various stress survivals associated with vacuolar function. Thus, pleiotropic phenotypes associated with defects at the vacuole are the common thread in this mutant collection. The fact that none of the *ENV* genes overlap with vacuolar protein sorting (*VPS*), vacuolar morphology (*VAM*), or monensin sensitivity (*MON*) genes suggests that they represent previously untapped and distinct vacuolar related gene functions. Accordingly, one third of the uncovered *ENV* genes are previously uncharacterized genes – *ENV7*, *ENV9*, *ENV10*, and *ENV11*. Genome-wide localization studies of GFP-tagged strains have reported Env7p at the vacuole membrane, Env9p in lipid particles, and Env11p in the nucleus [62]. To date, our preliminary microscopic and biochemical studies confirm localization of HA- and GFP- tagged Env7p to the vacuole membrane and Env11p to the nucleus (Manandhar, Oliveira, Ricarte, and Gharakhanian, unpublished results). Our bioinformatics analyses suggest that Env7p is a putative kinase with 29% identity with the human serine/threonine kinase STK16 [63]–[65], and *Env9p* is a putative oxidoreductase with 40% identity with the human retinol dehydrogenase RDH12, a gene associated with retinal degeneration [66]. The cellular functions of both human proteins remain unknown. Consistent with its nuclear localization, Env11p includes a putative zinc-finger DNA binding domain. These findings support a combination of enzymatic roles in vacuolar events by *ENV7* and *ENV9* gene products, as well as an indirect role through transcriptional regulation of other gene products by *ENV11*. Localization studies as well as functional and phylogenetic characterizations of novel *ENV* genes are in progress.

A persistent steady-state internal pool of p2CPY species could not be confirmed for *rps27bΔ* and *hog1Δ in western analyses* under normal or high salt suspended growth conditions . Since the same monoclonal antibody recognizes a pro-fragment containing species in patch immunoblots, the processing defect in *rps27bΔ* and *hog1Δ* may be in degradation of the ∼9 kDa pro-fragment following its cleavage from p2CPY. In repeated experiments, we did not detect such a species in 8–12% polyacrylamide gels as assessed by appropriate molecular weight markers (data not presented). Alternatively, the *env* phenotype in *rps27bΔ* and *hog1Δ* may be minimized in liquid cultures due to differences in vacuole dynamics, osmotic support or transcription/translation rates between liquid and semi-solid growth conditions. As the vacuole structurally and functionally responds to the environment, such phenotypic plasticity would not be inconsistent with the osmotic stress sensing role of Hog1p and the general translation role of the ribosomal subunit Rps27bp. Alternatively, their mutant strains may represent false positives of the screen at 2 out of total of 166 (1.2%) that were denoted as *env* or *vps* at the end of the secondary screen.

Additionally, we identified *ECM27* as a new *VPS* gene and established its deletion mutant as Class A with respect to vacuole morphology. *ECM27* was first uncovered in a screen for calcufluor hypersensitivity [67]. More recently, it was included in the top 20 genes whose deletion leads to CPY secretion in a genome-wide study [35]. Unlike most *vps* mutants, ecm27*Δ* does not exhibit a defect in retrograde trafficking as assessed in our halo assays. This difference may be why the gene was not picked up in *vps* screens and suggests that the involvement of Ecm27p in vesicular trafficking of CPY may be distinct from known Vps proteins. Bioinformatic analyses predict a signal peptide, multiple transmembrane domains, and homologies to sodium/calcium exchangers. The cellular function and localization of Ecm27p remain unknown.

### *ENV* genes and vacuole morphology

Microscopic studies of the *env* mutants reveal previously unreported vacuolar morphology functions for *PCP1*, *GET4/ENV8*, *PPE1*, *DCR2*, and *ENV11*. The null mutants of the genes have statistically significant increases or decreases in fragmented vacuoles relative to the isogenic wild type strain suggestive of *in vivo* vacuole fusion/fission defects. The products of *PCP1 and PPE1* genes are involved in mitochondrial events, and *pcp1Δ* mutants have been reported to exhibit fragmented mitochondria [39], [40]. Like the vacuole, mitochondria are dynamic organelles that engage in fusion and fission events. Furthermore, the product of *MAM3*, the third uncovered *ENV* gene to have a previously reported mitochondrial function, has been reported to be localized to the vacuole membrane [37]. Recently, phospholipid mediated signaling between mitochondria and vacuoles has been proposed [68], and mitochondrial contribution has been suggested in biogenesis of the autophagosome membrane in macroautophagy [69]. Together, these and our findings suggest that mitochondrial and vacuolar events may be deeply interconnected. Assessment of mitochondrial morphology in *env* mutants, particularly mutants exhibiting abnormal vacuolar morphology, may shed light on more global organellar fusion/fission gene functions.

Most interestingly, the novel mutant *env9Δ* exhibits lumenal vesicles within vacuoles – a previously unreported phenotype in vacuolar morphology studies. The *in vivo* lumenal vesicles are reminiscent of lumenal vesicles reported following *in vitro* homotypic fusion of isolated vacuoles [70]. Staining of the lumenal vesicle membranes with the endocytic and vacuole membrane marker FM4-64 suggests that their origin may be an endocytic compartment. Defects in breakdown of internalized vacuolar membranes following microautophagy (invagination of vacuole membrane), heterotypic fusion of late endosomal multivesicular bodies (MVBs) with vacuoles, or homotypic vacuole fusion would be consistent with the observed phenotype. Interestingly, high throughput genetic interaction studies report negative interactions between *ENV9* and *ENV10* as well as between *ENV9* and *GET4*/*ENV8* [71]. Assessment of CPY processing and vacuolar morphology in these double mutants may be especially instructive.

### *ENV* genes and stress survival

Several *env* mutants are sensitive to environmental stress conditions suggestive of defects in vacuolar stress survival. *hog1Δ* exhibits severe growth sensitivity to sodium consistent with the established role of its MAP kinase product in osmoregulation [50]. The mutant also exhibits growth sensitivities to potassium and low pH suggestive of HOG pathway mediated response under a range of stress conditions. A subset of *env* mutants exhibit tolerance or hypersensitivity to manganese. *mam3Δ* shows increased tolerance to manganese consistent with previous reports [37], while *sbe22Δ* and *rps27bΔ* exhibit no growth in its presence. The response to manganese does not apply generally to metals since no tolerance/hypersensitivity was observed with zinc. The two yeast manganese transporters, Smf1p and Smf2p, are ubiquitinated and directed to the vacuole for degradation through the MVB pathway at both physiological and toxic levels [72]–[74]. As such, tolerance or hypersensitivity to manganese in these mutants is likely due to perturbations of this vacuole-mediated down regulation.

Lastly, deletion of *RPS27B*, *MAM3* or *PCP1* results in rapamycin and caffeine hypersensitivities suggestive of signaling defects of TORC1, one of two conserved multi-subunit complexes of TOR kinase and responsible for temporal regulation of cell growth through upregulation of anabolic processes and inhibition of catabolic processes including vacuole/lysosome mediated autophagy [75]–[79]. In the latest studies in yeast and mammals, TORC1 appears predominantly localized to vacuoles and lysosomes [60], [80]–[82]. Intriguingly *MAM3* and *PCP1* are two genes with mitochondrial and vacuolar interconnections as discussed above. Thus, the *env* collection is enriched for defects in stress survival functions orchestrated at the vacuole.

Our results suggest that *ENV* genes affect events directly or indirectly at the vacuole. The uncovered genes affect vacuolar CPY processing at a post-endosomal stage, and the collection is enriched for heterotypic and/or homotypic fusion/fission and stress survival defects at the vacuole. The most significant contribution of this study is identification of four novel genes involved in vacuolar events– *ENV7*, *ENV9*, *ENV10*, *ENV11*. The *ENV* gene collection is poised to offer new insights into vacuolar events including fusion/fission dynamics and macromolecular degradation as well as possible connections to mitochondrial organization and function. Several *ENV* genes, including the novel *ENV7* and *ENV9*, appear to be orthologues of human genes with unknown cellular function. As such, their functional elucidation in yeast will likely contribute to further understanding of conserved lysosomal gene functions in health and disease.

## Materials and Methods

### Yeast media and strains

Yeast strains were grown on yeast extract-peptone-dextrose (YPD) or synthetic minimal (SM) media from Difco Chemicals (St. Louis, MO) and were prepared as described [83]. YPD or SM media were supplemented with selective components as specified. The MAT-α haploid *S. cerevisiae* deletion strain library, MAT-a haploids of the *env* and *vps* strains, and parental strains *BY4742* (MAT-α; *his3Δl*; *leu2Δ0*; *lys2Δ0*; *ura3Δ0*) and *BY4741* (MAT-a; *his3Δ1*; *leu2Δ0*; *met15Δ0*; *ura3Δ0*) were gifts from Dr. Greg Payne (UCLA). The collection of 4828 strains was developed by the *Saccharomyces* Genome Deletion project and contains 80% of the *Saccharomyces cerevisiae* genome, which was generated by PCR-based disruption of all open reading frames by chromosomal integration of a KanMX4 module through homologous recombination [84]. Mutants were stored in 15% glycerol in fifty-two 96-well plates at −80°C.

### Plasmids

PCR-based cloning was carried out to insert DNA sequences of previously uncharacterized *ENV* genes into the uracil-selectable CEN vector pRS316 [85]. Each ORF was amplified from BY4742 genomic DNA using custom-made forward (F1) and reverse (R1) primers (Operon; Huntsville, AL) that introduced restriction sites specified in parenthesis as follows:

pMSG370 was constructed using F1 (EagI) 5′-GGGGCGGCCGTGACGGTCAGAAAAGATTAGTCATT-3′; R1 (ClaI) 5′-GGGGATCGATTAATATGGGAGTGGGCAGCA-3′, which resulted in a 2.1-kb insert containing 608-bp upstream and 498-bp downstream of *YPL236C/ENV7*.

pTHG390 was constructed using F1 (EcoRI) 5′-GGGGGAATTCAAACAAGGGAATGGACGAGA-3′; R1 (XhoI) 5′-GGGGCTCGAGTAGACCACTGTCGTGCTTGG-3′, which resulted in a 2.1-kb insert containing 791-bp upstream and 298-bp downstream of *YOR246C/ENV9*.

pDOG3100 was constructed using F1 (EcoRI) 5′-GGGG GAATTCGAATAGGGGAGTGAGATCTG-3′; R1 (SacI) 5′-GGGGGAGCTCACATCATAATCAGCGGCTCG3′, which resulted in a 1.6-kb insert containing 508-bp upstream and 583-bp downstream of *YLR065C/ENV10*.

pLOG3110 was constructed using F1 (KpnI) 5′-GGGGGGTACCCACCAGTCACTGTACTTGAG-3′; R1 (SacI) 5′-GGGG GAGCTGCGTACCGGAAGTGTGTGATA-3′, which resulted in a 3.8-kb insert containing 626-bp upstream and 628-bp downstream of *YGR071C/ENV11*.

PCR reactions were carried out with Phusion DNA polymerase according to the manufacturer's instructions (New England Biolabs; Ipswich, MA). Recombinant plasmids were amplified in Top10 competent *E. coli* (Invitrogen; Carlsbad, CA). Preparation of plasmid DNA was carried out using Zyppy Plasmid Maxiprep and Midiprep Kits (Zymo Research; Orange, CA).

### Genomic screen for internal accumulation of p2CPY

The genomic screen is summarized in Figure 1A and consisted of sequential primary and secondary immunoblot assays that were modifications of an approach first described in Takahashi *et al.* [31]. In the primary screen, yeast strains were transferred from 96-well plates onto YPD plates with a pinning tool and incubated for 48 hours at 30°C. Cells were replica-plated onto nitrocellulose membranes and incubated at 30°C overnight. Following overnight incubation, membranes were washed with double-distilled water (ddH_2_O) and subjected to a 40 minute chase with 20 mg/ml cycloheximide (Sigma-Aldrich; St. Louis, MO) at 30°C. This translation inhibition step minimizes p1CPY and p2CPY species that are normal intermediates in the emergence of mature CPY in wild type cells. Membranes were washed three times for five minutes with ddH_2_O and were exposed to lysis buffer (0.1% SDS, 0.2 M NaOH, and 0.5% β-mercaptoethanol) for 40 minutes at room temperature. Membranes were then washed with ddH_2_O and blocked in a solution of tris buffered saline (50 mM Tris–HCl, pH 8.0, 150 mM NaCl) and 0.05% (v/v) Tween-20 in 5% (w/v) non-fat dry milk for 1 hour. Following blocking, membranes were probed with anti-proCPY specific mAb (Mab-4-H10-E6, U. Oregon) and were subjected to chemiluminescent autoradiography (Thermo Scientific; Rockford, IL).

In the secondary immunoblot assay, positive strains from the primary screen were replica plated onto two YPD plates and then transferred to two nitrocellulose membranes. Both membranes were incubated with 20 mg/ml cycloheximide for 40 minutes. One membrane was treated with lysis buffer for 40 minutes (lysed immunoblots) and the other was treated with ddH_2_O (non-lysed immunoblots) for 40 minutes. Membranes were then probed and developed as described. Following the secondary screen, deleted ORF's of uncovered *env* strains were confirmed by PCR using GoTaq (Promega; Madison, WI) and ORF-specific and KAN-cassette specific primers corresponding to those used for original library confirmations by the Saccharomyces Genome Deletion Project consortium (Operon; Huntsville, AL).

Following confirmation of deleted ORF's, *env* phenotype of each mutant was assessed in its MAT-a counterpart by immunodetection as described above. Only mutants whose *env* phenotype was confirmed in MAT-a were pursued further.

### Functional complementation of uncharacterized *env* mutants

To confirm linkage of the *env* phenotype to the deleted ORF's in the novel mutants *env7Δ*, *env9Δ*, *env10Δ*, and *env11Δ*, corresponding ORF's were reintroduced by means of transformation with recombinant CEN vectors pMSG370, pTHG390, pDOG3100, and pLOG3110, respectively, using Frozen EZ Yeast Transformation II kit (Zymo Research; Orange, CA). WT (BY4742) with parent and recombinant vectors containing each ORF were used as controls. Transformed cells were selected on SM media without uracil. Transformed cells were subjected to colony immunodetection as described in the primary screen, with slight modifications. Cells were patched onto SM-URA plates and incubated at 30°C for 72 hours. Following incubation, cells were replica-plated onto nitrocellulose membranes and incubated on YPD plates at 30°C overnight. Subsequent steps were as described for primary screen.

### Liquid invertase secretion assay

Quantitative liquid invertase assays were performed as described [86]. Secreted invertase is expressed as the percentage of extracellular invertase over total intracellular and extracellular invertase. Each strain was assayed a minimum of 3 times and statistical analysis was conducted using Minitab15 to determine if secretion was statistically different from wild type (Two-Sample *t*-Test, N = 3, confidence interval 95%, *p*<0.05).

### Halo pheromone assay

A single colony of GPY-1796 was resuspended in 1 mL YPD and added to 4 mL of 0.5% agar (Sigma; St. Louis, MO). Resuspended cells in YPD + agar solution were poured evenly onto a YPD plate and allowed to solidify for 10 minutes. Single colonies of *env* and wild type control strains were diluted to OD_660 nm_ = 0.26 and patched onto seeded plates using a sterile pinning tool. Plates were incubated at 30°C for 3 days. Halo assays were repeated a minimum of three times.

### Confocal microscopy

FM4-64 [N-(3-triethylammoniumpropyl)-4-(p-diethyla- minophenylhexatrienyl)pyridium dibromide] staining was performed as described [59]. Prior to staining, overnight cultures were diluted in YPD and harvested during mid-logarithmic phase (OD_600_ = 0.4–0.6). For costaining experiments, vital dye CellTracker Blue CMAC was added in addition to prescribed concentration of FM4-64 to a final concentration of 25 uM, followed by a 45 minute chase at 37°C. Stained cells were viewed with an Olympus Fluoview 1000 confocal laser scanning system mounted on an inverted microscope (Olympus IX-81) and a 100× oil immersion UPLSAPO (NA 1.4, WD 0.12 mm). The Argon ion (488 nm) and blue/red diode (405 nm/635 nm) lasers were used for image capturing. Images were equally zoomed to 4000× and were analyzed and processed with Adobe Photoshop CS5.

For every strain, the prominent phenotype was determined by scoring 300–400 non-budding cells in random fields. Raw data were subjected to statistical analysis using Minitab15 (Chi-squared Test, N = 5, confidence interval 95%, *p*<0.05).

### Growth assays in selective media and at high/low temperatures

YPD was supplemented with ions or drugs by adding NaCl, KCl, CaCl_2_, MnCl_2_, ZnCl_2_, rapamycin or caffeine to achieve concentrations specified in Figure 6 and Table 2. To prepare plates with pH of 2.5 or 8.0, HCl or NaOH was added to YPD, respectively. Monensin was resuspended in ethanol; SM Monensin plates and SM control plates containing ethanol were prepared as described [61]. Yeast colonies grown on YPD were suspended in sterile water to a concentration of OD_660_ = 0.26 (approximately 3.0×10^6^ cells/ml). Undiluted cells and serial 10-fold dilutions of up to 1∶1,000 were transferred using a pinning tool onto selective media. Plates were incubated at 30°C, and assessed for growth after 2 to 9 days. For temperature sensitivity assays, *env* and wild type strains were streaked onto pre-equilibrated YPD plates and incubated at 15°C for 7 days or 37°C for 3 days.

### Western blotting

Yeast cultures were grown at 30°C to logarithmic phase in YPD and whole cell lysates were prepared using Y-PER Yeast Protein Extraction Reagent, as directed by the manufacturer (Pierce; Rockford, IL). Protease inhibitor cocktail specific to yeast was added as directed by the manufacturer (Sigma; St. Louis, MO). Protein content was measured using Dc Protein Assay Kit (BioRad; Hercules, CA). Lysates were normalized for protein content and subjected to western analysis [87]. Membranes were blocked in a solution of tris buffered saline (50 mM Tris–HCl, pH 8.0, 150 mM NaCl) and 0.05% (v/v) Tween-20 in 5% (w/v) non-fat dry milk. Following blocking, membranes were probed with anti-proCPY specific mAb (Mab-4-H10-E6, U. Oregon) and loading control anti-hexokinase I specific pAB (Abcam; Cambridge, MA). Membranes were then subjected to chemiluminescent autoradiography (Thermo Scientific; Rockford, IL). Yeast strains exposed to high salt conditions were grown overnight at 30°C in YPD, washed once in YPD+0.75 M NaCl, and grown to logarithmic phase at 30°C in YPD+0.75 M NaCl. All subsequent steps were performed as described above.
